# Probing Our Built-in Calculator: A Systematic Narrative Review of Noninvasive Brain Stimulation Studies on Arithmetic Operation-Related Brain Areas

**DOI:** 10.1523/ENEURO.0318-23.2024

**Published:** 2024-04-04

**Authors:** Shane Fresnoza, Anja Ischebeck

**Affiliations:** ^1^Department of Psychology, University of Graz, 8010 Graz, Austria; ^2^BioTechMed, 8010 Graz, Austria

**Keywords:** arithmetic, mathematics, noninvasive brain stimulation, numerical processing, parietal cortex, triple code model

## Abstract

This systematic review presented a comprehensive survey of studies that applied transcranial magnetic stimulation and transcranial electrical stimulation to parietal and nonparietal areas to examine the neural basis of symbolic arithmetic processing. All findings were compiled with regard to the three assumptions of the triple-code model (TCM) of number processing. Thirty-seven eligible manuscripts were identified for review (33 with healthy participants and 4 with patients). Their results are broadly consistent with the first assumption of the TCM that intraparietal sulcus both hold a magnitude code and engage in operations requiring numerical manipulations such as subtraction. However, largely heterogeneous results conflicted with the second assumption of the TCM that the left angular gyrus subserves arithmetic fact retrieval, such as the retrieval of rote-learned multiplication results. Support is also limited for the third assumption of the TCM, namely, that the posterior superior parietal lobule engages in spatial operations on the mental number line. Furthermore, results from the stimulation of brain areas outside of those postulated by the TCM show that the bilateral supramarginal gyrus is involved in online calculation and retrieval, the left temporal cortex in retrieval, and the bilateral dorsolateral prefrontal cortex and cerebellum in online calculation of cognitively demanding arithmetic problems. The overall results indicate that multiple cortical areas subserve arithmetic skills.

## Significance Statement

The triple-code model (TCM) of numerical cognition remained an influential theory of number processing, including mental arithmetic. Here, we reviewed all noninvasive brain stimulation studies involving simple and complex symbolic arithmetic tasks and evaluated the three TCM assumptions based on the synthesized results. The review provides evidence for the first assumption that the bilateral intraparietal sulcus, which holds a magnitude code, engages in operations requiring numerical manipulations such as subtraction. The second assumption that the left angular gyrus subserves arithmetic fact retrieval, such as in multiplication, and the third assumption that the posterior superior parietal lobule engages in spatial operations were not supported. The results also provide evidence for the role of nonparietal areas in arithmetic abilities.

## Introduction

Dehaene's triple-code model (TCM) of numerical cognition draws a theoretical picture of number processing, including mental arithmetic, in the human brain (Dehaene et al., 2003). Based on different streams of research, such as experimental psychology, neuroimaging, and neuropsychological testing in humans, as well as single-unit recordings in monkeys, the TCM suggests three distinct codes for numbers represented in segregated neural populations in the parietal cortex.

### Assumptions of the TCM

The first TCM assumption is the presence of quantity representation or magnitude code at the bilateral intraparietal sulcus' horizontal segment (hIPS) for understanding and manipulating numerical quantities (Dehaene et al., 2003). Indeed, patients with IPS lesions have deficits in subtraction, an operation thought to require actual manipulation (online calculation) of numerical quantities but preserved knowledge of rote-learned arithmetic facts (Dehaene and Cohen, 1997; Cohen et al., 2000; Dehaene et al., 2003). The same holds for functional magnetic resonance imaging (fMRI) studies in healthy subjects, subtraction (with some exceptions of problems that can be stored in verbal memory such as 3 − 3 = 0), and two-digit addition and multiplication elicits left (Dehaene and Cohen, 1997; Kazui et al., 2000; Zago et al., 2001; Simon et al., 2002; Delazer et al., 2003, 2005; Kawashima et al., 2004; Ischebeck et al., 2006; Faye et al., 2019), right (Cohen et al., 2000; Kazui et al., 2000; Prado et al., 2011), and bilateral IPS activation (Lee, 2000; Menon et al., 2000; Kong et al., 2005; Zago et al., 2008; Andres et al., 2011; Klein et al., 2013a,b). The right hIPS is thought to be involved in forming spatial/structural diagrams that aid in spatial manipulation of numbers to obtain the solution (Miller et al., 2012).

The TCM also suggests a verbal code or verbal representation of numbers at the left angular gyrus (AG), where numerals are coded as words or sequences of words with lexical, phonological, and syntactical representations. The model predicted that simple or single-digit problems, such as multiplication, could be solved using operands (e.g., 2 × 5) transcoded into verbal code (two times five), which would elicit the rote memory (two times five equals ten) of this operation (Arsalidou and Taylor, 2011). Accordingly, left AG lesions impair simple addition, multiplication, and division but spare subtraction skills (Warrington, 1982; Lampl et al., 1994; Dehaene and Cohen, 1997; Cohen et al., 2000; Lee, 2000). In some cases, impaired multiplication with spared subtraction co-occurs with aphasia, indicating the dependency of multiplication on a language-based system of number processing (Dehaene and Cohen, 1997; Cohen et al., 2000; Dehaene et al., 2003). Multiplication engages the left AG because it is learned by rote memorization of multiplication tables in schools and, therefore, has strong associations between problems and solutions that are stored in long-term memory as arithmetic facts (Dehaene et al., 2003; Wassermann et al., 2008; Klein et al., 2013a,b; Faye et al., 2019). Single-digit additions (or subtraction) with a sum below 10 (e.g., 2 + 2 = 4) are also stored and retrieved from memory using the verbal code (but see for a different view). Increased left AG BOLD activity is observed during long-term memory retrieval (Menon et al., 2000; Stanescu-Cosson et al., 2000; Grabner et al., 2009; Jost et al., 2009; Prado et al., 2011; Klein et al., 2013a,b; De Visscher et al., 2015; Soylu and Newman, 2015) or when quantity-based processes shifted to automatic arithmetic fact retrieval (Ischebeck et al., 2006; Bloechle et al., 2016). Incorrect or “confusion” equations in which the proposed answer was true for a related operation (e.g., 9 × 6 = 15) also increased the left AG activation because they activate related arithmetic facts stored in verbal long-term memory (Grabner et al., 2013). The right AG activation was also reported and thought to be due to visuospatial attending processes crucial during fact retrieval (Grabner et al., 2009; Arsalidou and Taylor, 2011).

The model's third assumption is the presence of an Arabic code or visual representation of numbers at the bilateral posterior superior parietal lobule (PSPL). This code is thought to be used in accessing the other two systems through the visual modality and monitoring complex arithmetical calculations, such as those that require borrowing and carrying procedures, and magnitude code-independent operations, such as parity judgments (Dehaene et al., 1993; Wassermann et al., 2008). Indeed, there is significant bilateral PSPL activation during two-digit subtraction task performance or when two operations are performed instead of one (Lee, 2000; Menon et al., 2000; Andres et al., 2011). PSPL activation is also found to be problem size-, strategy-, and operands number-dependent (Menon et al., 2000; Stanescu-Cosson et al., 2000; Delazer et al., 2005; Zhou et al., 2007; Andres et al., 2011). Nonetheless, the PSPL's function is considered not specific for numerical computations since manual, visuospatial (e.g., saccade tasks), grasping, pointing, or linguistic tasks also activate it (Simon et al., 2002; Knops et al., 2009).

In recent years, theories that deviate from the original conception of TCM have been proposed (e.g., symbol-to-referent mapping hypothesis (Walsh, 2003), and updates of the model were suggested. This movement has been partly influenced by the “number neurons” discovery in the IPS and prefrontal cortex (PFC) of nonhuman primates (Nieder, 2016). The findings in animals are mirrored in human brain imaging studies; numerical processing robustly activates the inferior and superior parietal lobules, IPS, inferior and middle frontal gyrus, and cingulate gyrus (Arsalidou and Taylor, 2011; Nieder, 2016; Hawes et al., 2019). The nonparietal areas are now considered crucial for arithmetic proficiency as they serve domain-general processes, including working memory, episodic and semantic memory, and executive control (Garcia-Sanz et al., 2022).

### Noninvasive brain stimulation (NIBS)

NIBS techniques, such as transcranial magnetic stimulation (TMS) and transcranial electrical stimulation (tES) that can modulate neuronal excitability and induce neuroplastic effects, have rekindled interest in exploring the causal role of the parietal cortex for arithmetic processing. Traditionally, these methods were classified as either “facilitatory” or “inhibitory” based on their effect on motor-evoked potentials (MEPs) elicited by the single-pulse TMS stimulation of the contralateral motor cortex (Boroojerdi et al., 2001; Paulus et al., 2008; Ziemann et al., 2008; Klomjai et al., 2015). The MEP amplitude depends on motor pathway neuron depolarization with the electrical current induced by the strong time-varying magnetic field; hence, it is considered an index of corticospinal excitability. Single-pulse TMS has a short-lived effect; however, when applied repeatedly (repetitive TMS or rTMS) at low frequencies (<1 Hz) or high frequencies (>5 Hz), it can induce a build-up of sustained corticospinal excitability suppression and facilitation, respectively (Pascual-Leone et al., 1994; Chen et al., 1997; Wassermann et al., 1998; Houdayer et al., 2008). Similar effects are produced with repeated application of bursts containing three TMS pulses of high frequency (50 Hz) repeated within the theta frequency range (Huang et al., 2005). These patterned rTMS protocols are known as theta burst stimulation (TBS) and reduce cortical excitability when applied continuously for 20 or 40 s (continuous TBS) and increase cortical excitability when 2 s trains are repeated every 10 for 190 s (intermittent TBS). The aftereffects of rTMS mimic synaptic plasticity in animal models, specifically, namely, long-term depression (LTD) and long-term potentiation (LTP; Urban et al., 2002; Gersner et al., 2011). For instance, the long-lasting potentiation and depression of corticospinal excitability induced by TBS can be blocked by a *N*-methyl-d-aspartate (NMDA) receptor antagonist (Wankerl et al., 2010), as well as the long-lasting potentiation elicited by high-frequency rTMS (Schwenkreis et al., 2005). Meanwhile, low-frequency rTMS enhances the amplitude and duration of γ-aminobutyric acid (GABA-B)-mediated inhibitory postsynaptic potentials (Casula et al., 2014).

For tES, low-intensity (1–2 mA) electrical current is applied to the head using surface electrodes to modulate cortical neural activity (Reed and Cohen Kadosh, 2018). The most widely used method, transcranial direct current stimulation (tDCS), involves the continuous application of direct electrical currents, typically for 10 to 30 min. The concurrent effects of the bipolar tDCS stimulation of the motor cortex are akin to a “somatic doctrine” of membrane polarization. The inward current flow (injection of cations) from the anode electrode to the brain leads to the hyperpolarization of apical dendrites and then to the depolarization (excite) of the neuronal soma and axon hillock, whereas the outward current flow from the brain to the cathode causes the depolarization of apical dendrites (accumulation of negative charges on the outer surface of the membrane) and then the hyperpolarization (inhibition) of the neuronal soma (Nitsche et al., 2008; Stagg and Nitsche, 2011; Lefaucheur and Wendling, 2019). Meanwhile, the polarity-dependent aftereffects of tDCS on motor cortical excitability depend on a similar synaptic mechanism demonstrated in animals for glutamatergic LTP and LTD (Bear and Malenka, 1994). The anodal tDCS elicited potentiation, and the cathodal tDCS-elicited depression of MEP amplitudes beyond stimulation was shown to be NMDA- and calcium channel-dependent (Liebetanz et al., 2002; Nitsche et al., 2003, 2004).

There are also tES methods involving the application of rhythmic alternating current at a single frequency (transcranial alternating current stimulation or tACS) or at multiple changing frequencies ranging from 0.1 to 640 Hz and intensity (transcranial random noise stimulation or tRNS) or between 1 and 5 Hz (pulsed low-amplitude alternating electrical current or tPCS). For tACS, the online effect is attributed to the entrainment of endogenous oscillatory activity by the exogenously applied oscillating sinusoidal current in a frequency-specific (phase-to-phase locking) or cross-frequency (phase–amplitude coupling) manner (Antal and Paulus, 2013; Riddle et al., 2021). Entraining implies that the phase and/or frequency of the brain oscillation is modulated to follow external stimulation (Herrmann and Strüber, 2017). For instance, tACS applied at a fixed frequency (10 Hz) has no robust effect on corticospinal excitability (Antal et al., 2008) but increases it when applied at individual participants' alpha peak frequencies determined with electroencephalography recordings (Fresnoza et al., 2018). Meanwhile, the evidence indicates that plasticity mechanisms are sufficient to explain the aftereffects of tACS (Vossen et al., 2015) since an NMDA receptor antagonist suppresses the effect of 20 Hz tACS on corticospinal excitability and beta oscillations (Wischnewski et al., 2019).

In contrast to tACS, tRNS is applied at a wide frequency spectrum, between 0.1 and 100 Hz (low-frequency tRNS), between 101 and 640 Hz (high-frequency tRNS), and between 0.1 and 640 Hz (full-spectrum tRNS). For corticospinal excitability, low-frequency tRNS has no measurable effects, whereas high-frequency and full-spectrum tRNS increases it beyond the stimulation period (Terney et al., 2008; Moliadze et al., 2014). Studies have shown that the sodium channel blocker carbamazepine tends to inhibit corticospinal excitability after tRNS stimulation in humans (Chaieb et al., 2015). This supports the proposed mechanism behind tRNS online effects, which is the repetitive opening of the sodium channels, shown during the application of AC stimulation to rat hippocampal slices (Schoen and Fromherz, 2008). For the aftereffects, however, a plasticity mechanism is unlikely responsible because the partial NMDA receptor agonist d-cycloserine and the NMDA receptor antagonist dextromethorphan had no significant impact on the excitability increases after tRNS (Chaieb et al., 2015). Instead, the alternative proposed mechanism is stochastic resonance, where the introduction of white noise by tRNS boosts the synchronization of neural firing through the amplification of subthreshold oscillatory activity, which in turn reduces the amount of endogenous noise (Van Doren et al., 2014; Antal and Herrmann, 2016; Jaušovec and Pahor, 2017). Meanwhile, in tPCS, where the current is delivered through bilateral earlobe electrodes, the mechanism of action involves the modulation of the temporal cortex neuronal activity and frontotemporal functional connectivity by increasing interhemispheric coherence at low-range frequencies (Datta et al., 2013; Morales-Quezada et al., 2015).

In the study of higher-order cognitive functions such as arithmetic, NIBS can be applied “online” or simultaneously with task execution to modulate task-relevant ongoing neuronal activity. The injected current can serve as additional neural noise that can be beneficial if it is too weak to overwhelm the neural network and could actually boost the overall background activity the signal of interest is riding on. In some cases, however, the noise is so robust that it briefly interferes with the brain's ability to selectively disrupt the processing of distracting stimulus elements (Luber and Lisanby, 2014). In the case of tACS or rTMS, the stimulation could directly change neural dynamics by resetting, driving, or enhancing rhythmic neuronal firing that has functional relevance (Luber and Lisanby, 2014). For example, in the IPS, which is a part of the dorsal attention network involved in endogenous shifts of attention, individual alpha peak frequency-tuned tACS caused an attentional bias in a spatial cueing task (Kemmerer et al., 2022). This finding not only supports the functional role of alpha oscillations in visuospatial attention but also suggests that spatial attentional processing, which is also employed in solving arithmetic problems, is a highly probable process disrupted or enhanced by NIBS during online stimulation (Masson and Pesenti, 2014).

NIBS is also applied “off-line,” either before a task to induce up- or downregulation of intrinsic neuronal excitability that can affect task performance afterward, or in some cases, after a task to modulate consolidation processes in a learning paradigm (Hartwigsen and Silvanto, 2023). As the extension of effects beyond the stimulation period is attributed to neuroplasticity (except for tRNS), the potentiation and depression of synaptic strength would be the likely mechanism of arithmetic task performance improvement or impairment, respectively. An alternative mechanism put forward by Luber and Lisanby (2014) for the enhancing effect of TMS is called the “addition-by-subtraction” principle. This mechanism suggests that TMS might produce cognitive enhancement by disrupting or inhibiting an inessential or less essential but competing part of one or more functional brain networks involved in a task (Luber and Lisanby, 2014). For example, applying an inhibitory paradigm (e.g., 1 Hz rTMS) to brain areas contralateral to those relevant to the task might release interhemispheric inhibition.

### The present review

In the last decades, NIBS studies have provided us with a considerable amount of data to affirm and even challenge the TCM assumptions. However, the TCM has not been fully tested against the findings of all NIBS-arithmetic studies available to date because previous reviews focused on different aspects of numerical cognition (e.g., numerosity and magnitude representation), only one method (e.g., tES or TMS), or only one assumption (Sarkar and Cohen Kadosh, 2016; Schroeder et al., 2017; Faye et al., 2019; van Bueren, 2021a; Garcia-Sanz et al., 2022; Lazzaro et al., 2022). Therefore, this review comprehensively synthesized results from all NIBS studies involving simple and complex symbolic arithmetic tasks in healthy adults and patient groups to test the TCM assumptions.

## Materials and Methods

**Figure 1.**
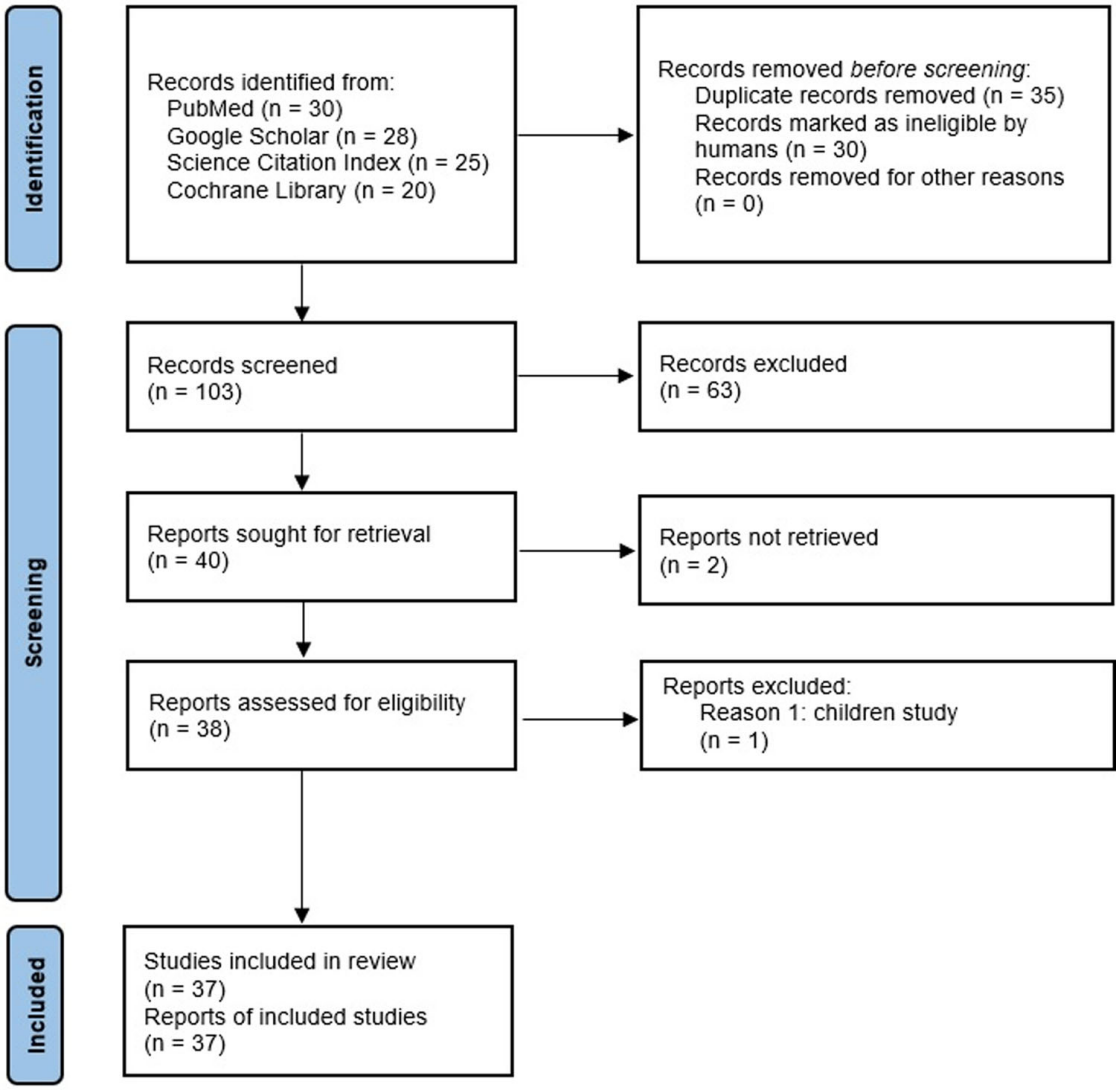
Number of included studies following the PRISMA flow diagram for systematic reviews and meta-analysis.

This review was conducted according to the accepted guidelines for the Preferred Reporting Items for Systematic Reviews and Meta-Analyses (Fig. 1; Page et al., 2021).

### Literature search

A systematic search was undertaken in the databases PubMed, Google Scholar, Science Citation Index Expanded, and the Cochrane Library to identify relevant publications from Jan. 2000 to Dec. 2022. Key search terms were: “brain stimulation,” “noninvasive brain stimulation,” “TMS,” “rTMS,” “tES,” “tDCS,” “ tACS,” “tRNS,” or “tPCS” combined with “arithmetic,” “arithmetic skills,” “mental arithmetic,” “problem-solving,” “arithmetic operations,” “mathematical problem-solving,” “number processing,” “calculation,” “addition,” “subtraction,” “multiplication,” and “division.” The reference lists of previous reviews and retrieved articles were also examined, titles and abstracts were screened for duplication, and full-text articles were retrieved to filter their eligibility based on the inclusion and exclusion criteria listed below.

### Eligibility criteria and quality assessment

For the selection of relevant articles, the study had to (1) include a sample of healthy subjects or patients solving arithmetic problems before and after, or during TMS, tDCS, tACS, tRNS, and tPCS stimulation of parietal and nonparietal brain areas; (2) be peer-reviewed; (3) be accessible (full-text) and written in English; (4) have a single- or double-blinded, randomized, sham-controlled, and crossover or parallel study design; (5) include participants ≥18 years of age; and (6) be carried out in accordance with the Helsinki Declaration. We excluded editorials, commentaries, meeting or conference abstracts, animal studies, and NIBS studies concerning nonarithmetical aspects of numerical cognition, which were discussed extensively elsewhere (Dehaene et al., 2003; Berch and Mazzocco, 2007; Wassermann et al., 2008; Looi and Cohen Kadosh, 2016; Sarkar and Cohen Kadosh, 2016; Schroeder et al., 2017; Faye et al., 2019; Hawes et al., 2019; van Bueren, 2021b). Eligible full-text articles were screened for study design, blinding method, number of participants and mean age, stimulator model, stimulation parameters, target cortical areas, localization methods, arithmetic and control tasks, task time (online vs off-line), and the main findings.

## Results

The initial search terms identified 103 potentially relevant articles published over the last 22 years (2000–2022). The screening process removed 63 articles, mainly NIBS studies concerning nonsymbolic arithmetic and magnitude tasks, as well as duplicate copies and studies with a nonretrievable full manuscript. One study in children (Looi et al., 2017) was excluded because the neurodevelopmental perspectives of arithmetic skills are not considered in the TCM, and the manner in which a child's brain engages in arithmetic might differ from that of an adult. For instance, greater PFC activation has been observed in younger children during arithmetic problem-solving, indicating greater cognitive and working memory demands during the early stages of skill acquisition (Rivera et al., 2005; Menon and Chang, 2021). In total, 37 original studies conducted with 1,219 participants met the inclusion criteria and were included in the review. Tables 1–3 provide an overview of each study's stimulation parameters. Thirty-three studies were conducted on healthy adults (*n* = 1,157) and four on patient groups (*n* = 62). Across the studies, the targets were the IPS (19), the AG (7), the PSPL (2), supramarginal gyrus (SMG), and nonparietal areas (24). The studies used various methods: tDCS (21), followed by TMS (8), tRNS (6), tACS (2), and tPCS (1). The primary outcome measures are reaction time (RT) and accuracy or error rates. None of the reviewed studies reported stimulation adverse or side effects. The following section describes the studies' main findings.

**Table 1.** Summary of stimulation parameters and results of TMS studies in healthy participants

	Study design	Number and mean age of participants	Target sites, localization methods (*)	Stimulator model and stimulation parameters	Tasks	Relevant findings
(Göbel et al., 2006)	Single-blind, between-subject design	Group 1 = 7 (24 years) Group 2 = 7 (25 years) *n* = 14	Bilateral areas between the IPS and AG and between the IPS and SMG *fMRI and BrainSight	Magstim Super Rapid (figure-of-eight coil) Intensity: 50–70% AMT Frequency: 10 Hz Duration: 20 s (5 s/block)	Two-digit addition (online) *-*No control task	RT slowing during left Ag + IPS and SMG + IPS stimulation
(Andres et al., 2011)	Single-blind, sham-controlled, within-subject design	*n* = 10 (21 ± 2 years)	Bilateral hIPS, bilateral PSPL, and vertex *fMRI and Polhemus	Magstim Rapid (figure-of-eight coil) Intensity: 65% MSO Frequency: 10 Hz Duration: 5.5 min (41.5 s/block)	One-digit subtraction and multiplication (online) *-*No control task	RT slowing in both operations during bilateral hIPS stimulation Bilateral PSPL stimulation has no significant effect on RTs
(Salillas et al., 2012)	Single-blind, sham-controlled, within-subject design	(exp.1) *n* = 12 (23 ± 3.53 years)	Session 1: right hIPS, right vIPS, or interhemispheric sulcus Session 2: left hIPS, left vIPS, or interhemispheric sulcus *Talairach coordinates, SoftTaxic Evolution Navigator system	Magstim 200 (figure-of-eight coil) Intensity: 110% of individual phosphine threshold Duration: max 3.36 min (33.65 s/block)	One-digit addition and multiplication (easy and difficult items, online) *-*No control task	RT slowing during bilateral hIPS for difficult addition and left hIPS for difficult multiplication RT slowing during bilateral vIPS for multiplication Addition and multiplication were impaired with SOAs of 250 and 300 ms
	Single-blind, within-subject design	(exp.2) *n* = 10 (24 ± 2.82 years)	Session 1: right hIPS and right vIPS Session 2: left hIPS and left vIPS *fMRI and BrainSight stereotaxic nauronavigator	Magstim Rapid (figure-of-eight coil) Intensity: 110% of individual phosphine threshold Duration: max 3.36 min (33.65 s/block)	One-digit addition and multiplication (difficult items, online) *-*No control task	RT slowing during bilateral hIPS for addition and during left hIPS for multiplication at 300 ms SOA RT slowing during right vIPS for multiplication at 150–250 ms SOAs
(Maurer et al., 2015)	Single-blind, sham-controlled, within-subject design	*n* = 12 (25 ± 1.7 years)	52 cortical spots in the right and left hemisphere (including the bilateral AG and PSPL) *fMRI and Nexstim eXimia NBS system	Nexstim (figure-eight coil) Intensity: 100% RMT Frequency: 5 Hz/5 pulses Duration: 5.4 s/spot (1.8 s/train)	One-digit addition, subtraction, multiplication, and division (online) *-*No control task	Left MFG: 45% ER for division Right AG: 40% ER for subtraction Left anterior STG: 35% for addition (8% on the right posterior STG) Left AG: 30% ER for multiplication Bilateral PSPL: no error recorder for all types of arithmetic tasks
(Montefinese et al., 2017)	Single-blind, sham-controlled, within-subject design	(exp.1) *n* = 10 (25.27 ± 4.79 years)	Session 1: right hIPS and vIPS or vertex Session 2: left hIPS and vIPS or vertex *fMRI and SofTaxi Optic	Magstim Rapid^2^ (figure-eight coil) Intensity: 65% of MSO Frequency: 10 Hz/4pulses Duration: 8.65 min (51.5 s/block)	Two-digit addition and subtraction (online) *-*No control task	RTs’ slowing is greater in addition than subtraction at the right hIPS
		(exp.2) *n* = 10 (28.11 ± 5.19 years)	Session 1: right AG and SMG Session 2: left AG and SMG *fMRI and SofTaxic Optic	Magstim Rapid^2^ (figure-eight coil) Intensity: 65% of MSO Frequency: 10 Hz/4pulses train Duration: 8.65 min (51.5 s/block)	Two-digit addition and subtraction (online) *-*No control task	RT slowing was greater for addition than subtraction with bilateral AG stimulation RT slowing was greater for subtraction than addition with the right than the left SMG stimulation
(Fresnoza et al., 2020)	Single-blinded, randomized, and sham-controlled design	*n* = 16 (26.25 ± 7.07 years)	Left hIPS, left AG, and vertex *fMRI and Localite TMS Navigator	MagPro X100 (figure-of-eight coil) Intensity: 110% AMT Frequency: 1 Hz Duration: 15 min	One-digit multiplication and complex subtraction (online and off-line) *-*Control task: Pegboard test	Left hIPS stimulation has no effect on the retrieval of answers for one-digit subtraction and multiplication but improved the online calculation of answers in both operations Left AG stimulation reduced RTs in multiplication (more than subtraction) problems regardless of strategy No significant effect on the control task
(Klichowski and Kroliczak, 2020)	Single-blinded, randomized, and sham-controlled design	*n* = 20 (20.9 ± 1.6 years)	Bilateral SMG *Brainsight® Frameless MNI ICBM 152 average brain template	Magstim® Rapid 2 Plus 1 (figure-of-eight coil) Intensity: 65% of the MSO Frequency: 10 Hz/3pulses train Duration: 2.33 min	Consumer-like arithmetic task involving multidigit mental subtraction (online) *-*Control task: multidigit mental addition	Significant RT slowing for discount calculations/mental subtraction during left SMG stimulation No significant effect on the control task

**Table 2.** Summary of stimulation parameters and results of TES studies in healthy participants

	Study design	Number and mean age of participants	Target sites, localization methods (*)	Stimulator model and stimulation parameters, current simulation software (*)	Tasks	Relevant findings
tDCS						
(Pope and Miall, 2012)	Single-blind, sham-controlled, between-subject design	Group 1 = 22 (21 years) Group 2 = 22 (20 years) Group 3 = 22 (21 years) *n* = 66	Right cerebellar hemisphere *Anatomical coordinates	Magstim DC Stimulator Plus Type: anodal, cathodal, or sham Intensity: 2 mA Electrode size: 25 cm^2^ Electrode montage: right cerebellar cortex–right deltoid muscle Duration: 20 min	PASST and PASAT tasks (off-line) *-*Control tasks: noun and verb reading, verb generation task	Cathodal tDCS increased the accuracy score and decreased the mean and standard deviation of RTs Verb generation learning increased after cathodal than anodal and sham tDCS
(Hauser et al., 2013)	Single-blind, sham-controlled, within-subject design	(exp.1) *n* = 20 (22.8 ± 3.1 years)	Bilateral IPS *10–20 EEG system	NeuroConn Type: anodal, cathodal, or sham Intensity: 1 mA Electrode size: 35 cm^2^, 100 cm^2^ Electrode montage: P3, P4–contralateral supraorbital area Duration: 25 min	Two-digit subtraction (off-line: before and after) *-*Control task: two-digit number comparison	Left IPS anodal tDCS reduced RT compared with sham No significant effect on the control task
		(exp.2) *n* = 16 (23.6 ± 2.4 years)	Right IPS *10–20 EEG system	NeuroConn Type: right anode or sham Intensity: 1 mA Electrode size: 35 cm^2^, 100 cm^2^ Electrode montage: P4–contralateral orbit Duration: 25 min	Two-digit subtraction (off-line: before and after) *-*Control task: two-digit number comparison	No significant effect on RT and accuracy rate No significant effect on the control task
(Klein et al., 2013a,b)	Single-blind, sham-controlled, within-subject design	*n* = 24 (26.2 ± 16.97 years)	Bilateral IPS *fMRI, 10–20 EEG system	NeuroConn Type: anodal, cathodal, sham Intensity: 1 mA Electrode size: 25 cm^2^, 100 cm^2^ Electrode montage: P3, P4–ipsilateral orbit Duration: 20 min *HD-Explore software (Soterix)	Two-digit addition problems (online) -Control task: color-word Stroop task	Bilateral anodal reduced and bilateral cathodal stimulation increased distractor distance effect No effect on the control task
(Clemens et al., 2013)	Single-blind, sham-controlled, within-subject design	*n* = 10 (43 ± 12.4 years)	Right AG *10–20 EEG system	NeuroConn Type: right anode or sham Intensity: 2 mA Electrode size: 35 cm^2^ Electrode montage: CP4–left orbit; Duration: 20 min	One-digit multiplication (off-line) *-*No control task	No significant effect on RT and task efficiency Significant increase in BOLD activity at the right AG
(Kasahara et al., 2013)	Single-blind, sham-controlled, within-subject design	*n* = 16 (21.1 ± 2.12 years)	Bilateral IPS *fMRI and Brainsight stereotaxic nauronavigator	NeuroConn Type: left anodal–right cathodal, left anodal F3/right cathodal, sham Intensity: 2 mA Electrode size: 35 cm^2^ Electrode montage: bilateral IPS Duration: 10 min	Two-digit and one-digit multiplication (online and off-line) *-*Control tasks: choice reaction task	Online left anodal–right cathodal tDCS shortened RTs in participants with left hemisphere dominance No significant effect on the control task
(Houser et al., 2014)	Single-blind, sham-controlled, between-subject design	Group 1 = 14 Group 2 = 15 Group 3 = 13 *n* = 42 (age not reported)	Left IPS *10–20 EEG system	Soterix Medical Type: left anode or sham Intensity: 1 mA or 2 mA Electrode size: 35 cm^2^ Electrode montage: P3–F3 Duration: 20 min	Complex arithmetic task (Kruskal–Wallis test) (off-line) -No control task	Calculation score was higher in the 1 mA group than the 2 mA and control groups
(Sarkar et al., 2014)	Double-blind, sham-controlled, between-subject design	Group 1 = 25 (21.4 ± 3.5 years) Group 2 = 20 (23.54 ± 3.11 years) *n* = 45	Bilateral DLPFC *10–20 EEG system	NeuroConn Type: left anodal–right cathodal, sham Intensity: 1 mA Electrode size: 25 cm^2^ Electrode montage: F3–F4 Duration: 30 min	One-digit addition, subtraction, multiplication, and division decisions task (online) -Control task: attentional network task (ANT)	Shortened RTs and decreased cortisol concentrations in high mathematics anxiety individuals Slow down RTs for individuals with low mathematics anxiety and prevented a decrease in cortisol concentration compared with sham stimulation No significant effect on ANT but impaired executive control in a flanker task
(Houser et al., 2015)	Single-blind, sham-controlled, between-subject design	Group 1 = 13 Group 2 = 10 Group 3 = 9 *n* = 32 (age not reported)	Left IPS *10–20 EEG system	Soterix Medical Type: left anode or sham Intensity: 1 mA or 2 mA Electrode size: 35 cm^2^ Electrode montage: P3–T4 Duration: 20 min	Complex arithmetic task (Kruskal–Wallis test, off-line) -No control task	Groups receiving 1 mA and 2 mA stimulation have faster calculation time compared with the control group
(Artemenko et al., 2015)	Single-blind, sham-controlled, within-subject design	*n* = 25 (23.28 ± 4.51 years)	Bilateral IPS *10–20 EEG system	NeuroConn Type: right or left anodal, right or left cathodal, sham Intensity: 1 mA Electrode size: 25 cm^2^, 100 cm^2^ Electrode montage: P3, P4–contralateral orbit Duration: 20 min *HD-Explore software (Soterix)	Two-digit addition (off-line) -Control task: color-word Stroop task	Right IPS anodal tDCS increases the carry effect but does not affect the target identity and distractor distance effect No significant effect on the control task
(Rütsche et al., 2015)	Single-blind, sham-controlled, within-subject design	*n* = 23 (21.78 ± 2.66 years)	Left AG *10–20 EEG system	NeuroConn Type: left anodal, sham Intensity: 1.5 mA Electrode size: 35 cm^2^,100 cm^2^ Electrode montage: P5, CP5–right orbit Duration: 30 min	Simple and complex addition and subtraction (off-line) -No control task	Anodal tDCS shortened RT latencies in large arithmetic problems (plus increased lower alpha ERD), decreased solution rates in small arithmetic problems (plus decreased theta band ERS)
(Grabner et al., 2015)	Double-blind, sham-controlled, between-subject design	Group 1 = 20 Group 2 = 20 Group 3 = 20 *n* = 60 (21.98 ± 2.99 years)	Left AG *10–20 EEG system	NeuroConn Type: left anodal, left cathodal, sham Intensity: 1.5 mA Electrode size: 35 cm^2^, 100 cm^2^ Electrode montage: P5–right orbit, CP5–right orbit Duration: 30 min *COMETS toolbox for MATLAB	Two-digit times one-digit multiplication, two-digit minus two-digit subtraction (off-line) -No control task	Cathodal tDCS prolonged RT for both operations, and anodal tDCS increased solution rates for subtraction in the learning phase Cathodal tDCS prolonged RT in both operations but only for trained problems in the performance phase
(Pope et al., 2015)	Single-blind, sham-controlled, between-subject design	Group 1 = 20 (22 ± 5 years) Group 2 = 20 (22 ± 2.3 years) Group 3 = 19 (21.4 ± 3.8 years) *n *= 59	Left DLPFC *10–20 EEG system	Magstim DC Stimulator Plus Type: left anodal, left cathodal, sham; Intensity: 2 mA Electrode size: 25 cm^2^ Electrode montage: F3–right deltoid muscle (a-left) Duration: 20 min	Complex addition (PASAT) and subtraction (PASST, off-line) -Control task: attention and mental fatigue rating with visual analog scale	Anodal tDCS reduced RTs, increased response accuracy, and decreased response latency variability between sessions only for PASST No significant effect on control tasks
(Plewnia et al., 2015)	Single-blind, sham-controlled, between-subject design	Group 1 = 14 Group 2 = 14 *n* = 28 (27.9 ± 9.3 years)	Left DLPFC *10–20 EEG system	NeuroConn Type: left anode or sham Intensity: 1 mA Electrode size: 35 cm^2^ Electrode montage: F3–right deltoid muscle Duration: 20 min	Adaptive PASAT (A-PASAT, online) -No control task	Anodal tDCS improved calculation speed on A-PASAT Anodal tDCS increases cognitive control as indicated by a reduction of attentional biases induced by emotionally salient stimuli
(Gill et al., 2015)	Single-blind, sham-controlled, within-subject design	(exp.1) *n* = 11 (21.8 ± 2.7 years)	Left DLPFC *10–20 EEG system	Magstim DC Stimulator Plus Type: left anode or sham Intensity: 2 mA Electrode size: 25 cm^2^ Electrode montage: F3–right supraorbital area (a-left) Duration: 20 min	Adaptive PASAT (A-PASAT) (off-line) Training task: three-back letter task -No control task	Anodal tDCS during the three-back letter task significantly improved accuracy on A-PASAT compared with sham
		(exp.2) *n* = 11 (19.8 ± 1.5 years)	Left DLPFC *10–20 EEG system	Magstim DC Stimulator Plus Type: left anode or sham Intensity: 2 mA Electrode size: 25 cm^2^ Electrode montage: F3–right supraorbital area Duration: 20 min	Adaptive PASAT (A-PASAT) (off-line) Training task: one-back letter task -No control task	Anodal tDCS during the one-back letter task does not affect A-PASAT performance
(Hauser et al., 2016)	Single-blind, sham-controlled, between-subject design	Group 1 = 20 (22.4 ± 3 years) Group 2 = 20 (22.4 ± 3.6 years) *n* = 40	Left IPS *10–20 EEG system	NeuroConn Type: left anode–right cathode, sham Intensity: 1 mA Electrode size: 35 cm^2^, 50 cm^2^ Electrode montage: CP5–P5, Fpz3–AF8 Duration: 30 min *COMETS toolbox for MATLAB	Two-digit minus two-digit subtraction (online) -No control task	Repeated problems activate the bilateral AG and medial plus lateral prefrontal cortices Novel problems activate the bilateral IPS and dorsomedial prefrontal cortex tDCS decreased right inferior frontal cortex activation, while participants solved novel (compared with repeated) problems No significant differences in arithmetic task performance were observed between stimulation conditions
(Luft et al., 2017)	Single-blind, sham-controlled, between-subject design	Group 1 = 20 (24.05 ± 4.7 years) Group 2 = 20 (23.3 ± 3.9 years) Group 3 = 20 (22.7 ± 3.19 years) *n* = 60	Left DLPFC *10–20 EEG system	Starstim Type: left anode, cathode, and sham Intensity: 1 mA Electrode size: 1 cm radius Electrode montage: F3–three return electrodes (T7, Cz, and Fp2) Duration: 30 min *StimWeaver (Neuroelectrics)	Easy and difficult matchstick arithmetic problems (off-line) -No control task	Cathodal tDCS improved solution rate and success index for complex trials
(Mosbacher et al., 2020)	Double-blind, sham-controlled, between-subject design	Group 1 = 21 (22.8 ± 3.4 years) Group 2 = 21 (27.5 ± 5.4 years) Group 3 = 20 (26.6 ± 5 years) *n* = 62	Left IPS and left DLPFC *10–20 EEG system	NeuroConn Type: left anode, sham Intensity: 1 mA Electrode size: (anode) 9 cm^2^, (cathode) 35 cm^2^ Electrode montage: F3–RO, P3–RO Duration: 25 min	One-digit and two-digit additions and subtractions (online and off-line) -Control task: two-back task	Left IPS and DLPFC anodal tDCS has no significant effect on calculation time and accuracy for one- and two-digit addition and one-digit subtraction Left DLPFC anodal tDCS significantly reduced calculation time for two-digit subtraction No stimulation-related effects on theta and low and high alpha ERS/ERD No significant effect on the control task
tACS						
(Mosbacher et al., 2021)	Double-blind, sham-controlled, between-subject design	Group 1 (sham) = 19 (22.4 ± 3.6 years) Group 2 (DLPFC tDCS) = 20 (21.8 ± 2.7 years) Group 3 (PPC tDCS) = 18 (22.3 ± 3.4 years) Group 4 (alpha tACS DLPFC) = 20 (23.8 ± 4.9 years) Group 5 (alpha tACS PPC) = 20 (22.5 ± 3.2 years) Group 6 (theta tACS DLPFC) = 20 (21.8 ± 3.7 years) Group 7 (theta tACS PPC) = 20 (23.0 ± 4.6 years) *n* = 137	Left DLPFC and left PPC *10–20 EEG system	NeuroConn -tDCS Intensity: 1 mA, sham Electrode size: (anode) 9 cm^2^, (cathode) 35 cm^2^ Electrode montage: F3–RO, P3–RO Duration: 25 min -tACS Intensity: 1.5 mA maximum Electrode size: (anode) 9 cm^2^, (cathode) 35 cm^2^ Electrode montage: F3–left shoulder, P3–left shoulder Frequency: individual alpha or theta Duration: 25 min	one-digit and two-digit pound arithmetic task (online) -No control task	Theta tACS of the DLPFC or PPC improved novel arithmetic fact learning Alpha tACS and anodal tDCS of the DLPFC or PPC have not affected arithmetic fact learning No effect on the acquisition and training of the novel procedure No significant stimulation-related changes in ERS/ERD pattern
(Zhang et al., 2022)	Single-blind, sham-controlled, within-subject design	*n* = 20 (27.5 ± 4 years)	Right MFG and IPS *10–10 system	Starstim 8 (Neuroelectics) Intensity: 1 mA Electrode size: 1 cm radius Electrode montage: 2 × 1 (Fp2–F4–Fz, Pz–P4–O2) Frequency: slow theta (4 Hz) and individual subject tuned theta, sham at 6 Hz Duration: 25 min *Starstim 8	Visuospatial WM task (online) *-*Control task: mental rotation (MR), one-digit and two-digit addition and subtraction (online)	In-phase, individually tuned theta tACS improved vsWM and MR and increased frontoparietal phase synchronization Fixed, slow (4 Hz) theta did not show any behavioral or electrophysiological effects No significant effects on arithmetic tasks
tRNS						
(Cappelletti et al., 2013)	Double-blind, sham-controlled, between-subject design	*n* = 40 (25.8 ± 12 years)	Bilateral IPS and bilateral M1, bilateral IPS and bilateral M1 *10–20 EEG system	NeuroConn Intensity: 1 mA Electrode size: 35 cm^2^ Frequency: 0–250 Hz, sham Electrode montage: P3–P4 Duration: 20 min	Training task: numerosity discrimination task (online) Testing task: space and time discrimination task; one-digit addition, subtraction, and multiplication; two-digit addition and subtraction; two-digit and one-digit multiplication (off-line) *-*Control tasks: number Stroop, attention network, and face sequential matching task	Numerosity discrimination improved and stable until 16 weeks posttraining Transfer effect for space and time discrimination task No transfer effect for arithmetic task No transfer effect for the control tasks
(Cappelletti et al., 2015)	Double-blind, sham-controlled, between-subject design	Group 1 = 30 (24.4 years) Group 2 = 30 (65.5 years)	Bilateral IPS and bilateral M1 *10–20 EEG system	NeuroConn Intensity: 1 mA Electrode size: 35 cm^2^; Frequency: 0.1–640 Hz, sham Electrode montage: P3–P4 or C3–C4 Duration: 20 min	Training task: numerosity discrimination task (online) Testing task: space and time discrimination task; one-digit addition, subtraction, and multiplication (off-line) -Control tasks: number and word Stroop, attention network, face sequential matching, and Navon tasks	Numerosity discrimination improved across age groups and was stable until 16 weeks posttraining Age-dependent effect on the transfer effect to related task: improve and impaired space and time discrimination in young and old participants, respectively Number and word Stroop, ANT, and arithmetic performance did not differ across stimulation groups Training has no effect on the Navon task in the elderly group and visual pattern recognition in the young group
(Snowball et al., 2013)	Double-blind, sham-controlled, between-subject design	Group 1 = 13 (20.92 ± 2.10 years) Group 2 = 12 (21.42 ± 3.23 years) *n* = 25	Bilateral DLPFC *10–20 EEG system	NeuroConn Intensity: 1 mA Electrode size: 25 cm^2^ Duration: 20 min Frequency: 100–600 Hz, sham Electrode montage: F3–F4 Duration: 20 min	Arithmetic algorithm: one single-digit and one double-digit operand calculation task, two-digit-operand for drill task (online training) -Control task: mental rotation task and attention network test	Improved calculation and drill learning rates after 5 d of training Reduced peak amplitude and latency of hemodynamic responses at the left DLPFC at the end of the training Improvement only in calculation task after 6 months Decreased peak latency of hemodynamic responses for calculation tasks No effect on the control task
(Pasqualotto, 2016)	Double-blind, sham-controlled, between-subject design	Group 1 = 18 (21.2 ± 3.1 years) Group 2 = 18 (21.3 ± 2.6 years) Group 3 = 18 (22.0 ± 4.4 years) *n* = 54	Bilateral IPS and bilateral DLPFC *10–20 EEG system	NeuroConn Intensity: 1 mA, sham Electrode size: 25 cm^2^ Frequency: 100–600 Hz Electrode montage: F3–F4, P3–P4 Duration: 20 min	Two-digit subtraction verification task (online) -Control task: word classification task	Testing phase: frontal and parietal stimulation improved RT but had no significant effect on accuracy Retesting: frontal and parietal stimulation improved the accuracy No significant effect on the control task
(Popescu et al., 2016)	Double-blind, sham-controlled, between-subject design	Group 1 = 16 (21.94 ± 3.55 years) Group 2 = 16 (22.81 ± 3.23 years) *n* = 32	Bilateral DLPFC (Days 1–3), bilateral posterior parietal cortex (including the IPS) on Days 4–5 *10–20 EEG system	Magstim DC Stimulator Plus Intensity: 1 mA Electrode size: 16 cm^2^ Frequency: 100–640 Hz, sham Electrode montage: F3–F4, P3–P4 Duration: 20 min	One-digit and two-digit addition, subtraction, and multiplication (online and off-line) -Control tasks: Wechsler Individual Achievement Test (WIAT) before training, digit-span task, and ANT after Day 1 and Day 5 of the training	Training phase: tRNS shortened RTs in calculation tasks only in difficult trials Testing phase: tRNS improved the accuracy of calculation tasks only for easy and new trials No significant effect on the control tasks
(Bieck et al., 2018)	Single-blind, sham-controlled, within-subject design	*n* = 48 (23.48 ± 3.30 years)	Bilateral IPS and bilateral DLPFC *10–20 EEG system	NeuroConn Intensity: 1 mA Electrode size: 25 cm^2^ Frequency: 100–640 Hz, sham Electrode montage: F3–F4, P3–P4 Duration: 20 min *HD-Explore software (Soterix)	Two-digit addition (online) -Control task: color-word Stroop task	Parietal stimulation did not modulate the distractor distance, carry, and target identity effect Frontal stimulation increases the distractor distance effect No significant effect on the control task
(Krause et al., 2019)	Double-blind, sham-controlled, between-subject design	(exp 1.) *n* = 1 (46 years)	Bilateral DLPFC *10–20 EEG system	Starstim (Neuroelectrics) Intensity: 1 mA Electrode size: 25 cm^2^, circular Frequency: 0.1–500 Hz, sham Electrode montage: F3–F4 Duration: 20 min	Complex multiplication algorithm (online) -No control task	No improvement in calculation performance
		(exp. 2) *n* = 6 (age not reported)	Bilateral DLPFC *10–20 EEG system	Starstim (Neuroelectrics) Intensity: 1 mA Electrode size: 25 cm^2^, circular Frequency: 0.1–500 Hz, sham Electrode montage: F3–F4; Duration: 20 min	Two-digit by two-digit, three-digit by two-digit, three-digit by one-digit, four-digit by one-digit multiplication (online) -No control task	tRNS impaired accuracy compared with sham tRNS and sham have a comparable effect on RTs
tPCS						
(Morales-Quezada et al., 2015)	Double-blind, sham-controlled, between-subject design	Group 1 = 15 (30.53 ± 7.59 years) Group 2 = 15 (28.40 ± 5.15 years) *n* = 30	Bilateral temporal cortex *Anatomical	BrainGear Intensity: 2 mA Frequency: 1–5 Hz, sham Electrode montage: bilateral earlobes Duration: 20 min	One-digit, two-digit and multi-digit subtraction (off-line) Control task: Balloon Analog Risk Task, Stroop color-word task (off-line)	No significant effect on the simple and complex subtraction task performance compared with sham No significant effect on the control task and physiological measurements (e.g., heart rate and electrodermal activity)

**Table 3.** Summary of stimulation parameters and results of NIBS studies in patients

	Study design	Number and mean age of participants	Target sites, localization method (*)	Stimulator model and stimulation parameters, current simulation software (*)	Task	Relevant findings
(Ille et al., 2018)—TMS	Single-blind, within-subject design	*n* = 26 (62.42 ± 15.57 years)	52 cortical spots in the right and left hemisphere *MRI and eXimia nTMS system	Nexstim (figure-of-eight coil) Intensity: 100% RMT Frequency: 5 Hz/10 pulses Duration: 10.8 s/spot (1.8 s/train)	nrTMS mapping: one-digit addition, subtraction, multiplication, and division (online) Pre- and postoperative testing: number-processing and calculation task (NPCT) -No control task	Highest error rates: Addition: left AG and posterosuperior frontal gyrus Subtraction: right middle frontal and postcentral gyrus, left middle and superior frontal and temporal gyrus, left dorsolateral occipital gyrus Multiplication: left middle frontal gyrus Division: right middle temporal and left inferior, middle and superior frontal gyrus, left ventral and dorsal precentral gyrus, left dorsal and middle postcentral gyrus, left SPL, left AG, and dorsolateral occipital gyrus nrTMS arithmetic-positive cortical site resection correlates with the worsening of arithmetic skills in NPCT
(Iuculano and Cohen Kadosh, 2014)—tDCS	Single-blind, between-subject design	*n* = 2 (29.5 ± 4.95 years)	Bilateral PPC *10–20 EEG system	NeuroConn Type: right anode–left cathode, left anode–right cathode Intensity: 1 mA Electrode size: 9 cm^2^ Electrode montage: P3–P4 Duration: 20 min	Learning task: artificial digits (Gibson figures, off-line) Testing task: numerical Stroop and number line task (off-line) -No control task	Left anodal–right cathodal stimulation improved performance in numerical Stroop and number line task
(Vanderhasselt et al., 2015)—tDCS	Double-blind, sham-controlled, between-subject design	Group 1 = 19 (46.29 + 10.67 years) Group 2 = 14 (41 + 11.4 years) *n* = 33	Bilateral DLPFC *10–20 EEG system	Chattanooga Ionto device Type: left anode –right cathode or sham Intensity: 2 mA Electrode size: 25 cm^2^ Electrode montage: P3–P4 Duration: 30 min	Adaptive PASAT (A-PASAT, online) Ruminative Response Scale -No control task	DLPFC tDCS reduced maladaptive ruminative but did not affect depression severity and arithmetic performance
(Sreeraj et al., 2019)—tDCS	Single-case report	*n* = 1 (43 years)	Left DLPFC (Phase 1) *10–20 EEG system	TCT stimulator Type: left anode Intensity: 1 mA Electrode size: 35 cm^2^, 54 cm^2^ Electrode montage: F3–right supraorbital area Duration: 20 min	Categorical fluency test (CFT), digit span task, trail-making test, spatial span, letter–number sequencing, COWA, and Mini-Mental State Examination (off-line)	Improved CFT and controlled oral word association test (COWA) performance
			Left PPC (Phase 2) *10–20 EEG system	NeuroConn Type: left anode Intensity: 1 mA Electrode size: 35 cm^2^ Electrode montage: P3–left supraorbital area Duration: 20 min	Number fluency, numerosity, gestalt and constructional perception, mental rotation task, digit span test, one-digit and three-digit addition, subtraction, multiplication, and division (off-line)	Improved performance in all tasks, except single-digit multiplication and division, and all complex arithmetic

### Effects of IPS stimulation on arithmetic performance

The TCM proposes the engagement of bilateral IPS whenever a task requires access to number magnitude representation and calculation procedure (Dehaene et al., 2003). This assumption seems to be valid since RTs for subtraction problems are prolonged by right or left hIPS 10 Hz rTMS (Andres et al., 2011; Montefinese et al., 2017) and for difficult addition problems by single-pulse TMS (Salillas et al., 2012). 10 Hz rTMS and single-pulse TMS of the right or left ventral portion of the IPS (vIPS) also prolonged RTs for addition and subtraction and difficult multiplication problems, respectively (Salillas et al., 2012; Montefinese et al., 2017). Notable is the hemispheric interference asymmetry. In the Montefinese study, more interference was found for right than left hIPS stimulation for addition and subtraction and stronger for right hIPS than right vIPS stimulation for addition. In the Salillas study, interference for multiplication, which is related to problem size, was also stronger for the right vIPS. Left hIPS 1 Hz rTMS did not impair subtraction task performance, also indicating the right hemispheric dominance for online calculation (Fresnoza et al., 2020).

Results from tES studies are also broadly consistent with the first assumption. For example, participants who received 1 and 2 mA left IPS anodal tDCS have shorter calculation times and higher scores than the control group (Houser et al., 2014, 2015). Left but not bilateral IPS anodal tDCS also shortened RTs for a two-digit subtraction task and increased accuracy in a magnitude comparison task (Hauser et al., 2013). Meanwhile, bilateral IPS tDCS with left anode–right cathode montage (but not the reverse montage) shortened RTs for two-digit times one-digit multiplication problems (e.g., 64 × 7) in participants with left calculation dominant hemisphere (Kasahara et al., 2013). In a two-digit addition verification task, simultaneous bilateral IPS anodal or cathodal stimulation also reduced and increased the distractor distance effect, respectively (Klein et al., 2013a,b), while right IPS anodal but not cathodal stimulation prolonged latency increases for carry operations on the same task (Artemenko et al., 2015).

The results of tRNS studies, on the other hand, point to a beneficial effect of IPS stimulation for enhancing arithmetic proficiency through learning rather than facilitating actual numerical task performance. As proof of this, bilateral IPS high-frequency tRNS shortened RTs and improved accuracy in two-digit subtraction verification tasks during testing and retesting regardless of problem novelty (Pasqualotto, 2016). Coupling training with bilateral IPS full-spectrum tRNS also stabilized the training-induced performance improvement, although the transfer effect was somewhat limited to related tasks or modalities (Cappelletti et al., 2013, 2015; Popescu et al., 2016). In the Popescu study, 5 d training with drill and calculation problems coupled with bilateral PFC high-frequency tRNS (first 3 d) and IPS high-frequency tRNS (last 2 d) consequently improved RT's latency and distribution for calculation problems in the difficult condition during training and accuracy in the easy and new conditions during testing. In the Cappelletti studies, 5 d training of numerosity discrimination concurrent with bilateral IPS full-spectrum tRNS led to a long-term (16 weeks) improvement in the training task across age groups. However, the training had an age-dependent effect on the transfer effect to untrained related tasks: space and time discrimination was improved and impaired in young and old participants, respectively. Meanwhile, training did not significantly affect untrained and unrelated tasks, including simple and complex symbolic arithmetic tasks.

There are also results at odds with the first assumption of the TCM. Some studies showed an impairment of arithmetic fact retrieval under IPS stimulation: longer RTs were observed in one-digit multiplication during left but not right hIPS single-pulse TMS (Salillas et al., 2012) and in two-digit addition during 10 Hz rTMS applied between the left AG and the posterior part of the IPS (Göbel et al., 2006). Meanwhile, in another study, rote-learned one-digit subtraction and multiplication were unaffected by left hIPS 1 Hz rTMS (Fresnoza et al., 2020). Another result that is at odds with the first assumption of the TCM is that left PPC theta tACS decreased calculation times in novel fact-learning problems, as the theta frequency band indexes fact retrieval from memory (Mosbacher et al., 2021). Other results in contrast to the first assumption are the absent effects of right IPS anodal tDCS on two-digit subtraction and magnitude comparison (Hauser et al., 2013, 2016) and the absent effects of left IPS anodal tDCS on one-digit subtraction and one and two-digit additions, as well as two-back task performance (Mosbacher et al., 2020), and of left PPC alpha tACS and anodal tDCS on the training and acquisition of a novel arithmetic procedure and novel arithmetic facts (Mosbacher et al., 2021). Bilateral IPS high-frequency tRNS also failed to modulate target identity, distractor distance, and carry-over effects in a two-digit addition verification task (Bieck et al., 2018).

### Effects of AG stimulation on arithmetic performance

The second TCM assumption states that the left AG is involved in the verbally mediated retrieval of symbolic number knowledge and arithmetic facts (Dehaene et al., 2003; Wassermann et al., 2008; Sokolowski et al., 2017). This is corroborated by several results: 5 Hz rTMS of the left AG or at an area very close to it (left anterior superior temporal gyrus) evoked the highest error rate for single-digit multiplication (30%) and one-digit addition (35%), respectively (Maurer et al., 2015). Furthermore, left AG 1 Hz rTMS prolonged RTs for subtraction problems often solved by retrieval (e.g., 14 − 7), simple multiplication problems, and online calculation (Fresnoza et al., 2020). There was also no effect of right AG anodal tDCS on one-digit multiplication verification task performance (Clemens et al., 2013). However, several results indicate involvement of the left or bilateral AG in complex calculations: right AG 5 Hz rTMS increased the error rate for simple addition problems (Maurer et al., 2015), 10 Hz rTMS to either the right or left AG prolonged RTs for complex addition and subtraction problems (Montefinese et al., 2017), and 10 Hz rTMS between the left AG and IPS prolonged RTs for double-digit addition (Göbel et al., 2006). Although Göbel et al. attributed the effect to memory retrieval difficulties, this is unlikely because their participants reported mentally calculating the solutions. In addition, Grabner et al. (2015) showed that left AG cathodal tDCS coupled with arithmetic fact training prolonged RTs for complex multiplication and subtraction that persisted over 24 h but only for trained problems. In the same study, left AG anodal tDCS and training improved subtraction but not multiplication learning (Grabner et al., 2015). Left AG anodal tDCS also improved response latencies in large operand addition problems assumed to be solved by calculation (two-digit/two-digit carry problem with addends ranging from 12 to 29) but decreased solution rates for small operand addition problems assumed to be solved by memory retrieval (one-digit/one-digit problems with addends between 2 and 8; Rütsche et al., 2015).

### Effects of PSPL stimulation on arithmetic skills

The third TCM assumption suggests a supportive role for the PSPL for the other two brain areas because it is assumed to hold the visual representation of numbers (Dehaene et al., 1993; Wassermann et al., 2008). However, the two rTMS studies that directly stimulated the bilateral PSPL (Table 1) failed to demonstrate a significant effect on single-digit addition, subtraction, multiplication, and division task performance (Andres et al., 2011; Maurer et al., 2015).

### Effects of SMG and nonparietal area stimulation on arithmetic skills

Dehaene and colleagues also outlined domain-general processes interfacing with numerical cognition, such as executive function, spatial attention, and working memory (Arsalidou and Taylor, 2011; Schroeder et al., 2017; Siemann and Petermann, 2018). Against this background, several studies that targeted nonparietal and parietal areas outside those postulated by the TCM enhanced arithmetic proficiency.

#### SMG

The neuroimaging literature associated the right SMG with spatial working memory and attention shifting, particularly for cognitively demanding arithmetic tasks (Silk et al., 2010; Abd Hamid et al., 2011; Faye et al., 2019). Indeed, 10 Hz rTMS elicited a lateralized (right SMG > left SMG) detrimental effect on two-digit subtraction but not addition problems, which the authors attributed to the former being relatively less automated and more difficult to solve than the latter (Montefinese et al., 2017). Consistently, compared with all cortical targets, the highest error rate (11%) for single-digit subtraction was elicited at the right posterior SMG by 5 Hz rTMS (Maurer et al., 2015). Meanwhile, for the left SMG, some neuroimaging studies suggested a role in arithmetic fact retrieval (Faye et al., 2019). Ten Hz rTMS of the anterior part of the left SMG prolonged RTs in a consumer-like discount calculation task involving multidigit mental addition or subtraction problems (Klichowski and Kroliczak, 2020), while when applied between the left SMG and anterior IPS, it impaired two-digit addition task performance (Göbel et al., 2006).

#### PFC

Arithmetic tasks involving procedural strategies recruit the frontal lobe, particularly the dorsolateral region of the PFC (DLPFC; Grabner et al., 2009; Arsalidou and Taylor, 2011; Hawes et al., 2019; Mosbacher et al., 2020). Interestingly, it is also one of the core brain regions showing elevated activity in calculation prodigies (Pesenti et al., 2001; Krause et al., 2019). However, instead of a specialized function in arithmetic, the PFC is ascribed a role in more general functions such as top-down attention, executive functions, and working memory, the cognitive space where number representations can be stored and manipulated (e.g., choice of strategy and planning; Arsalidou and Taylor, 2011; Klein et al., 2013a,b, 2016; Matejko and Ansari, 2015; Schroeder et al., 2017; Siemann and Petermann, 2018; Hawes et al., 2019).

A consistent finding of the studies reviewed here was improved arithmetic proficiency by unilateral or bilateral tES stimulation of the dorsolateral region of the PFC, particularly for cognitively demanding tasks (Table 2). For instance, left PFC anodal tDCS increased accuracy, shortened RTs, and decreased RT variability on the Paced Auditory Serial Subtraction Task (PASST) but had no effect on the less challenging Paced Auditory Serial Addition Task (PASAT; Pope et al., 2015). Meanwhile, in the more difficult adaptive PASAT (A-PASAT), left PFC anodal tDCS improved calculation speed and reduced emotionally salient stimuli-elicited attentional biases (Plewnia et al., 2015), as well as improved calculation accuracy when coupled with a complex three-back but not one-back training task (Gill et al., 2015). Left PFC anodal tDCS also shortened RTs for two-digit subtraction problems but had no effect on single-digit addition and subtraction problems, as well as on easy two-back working memory tasks (Mosbacher et al., 2020). Similarly, high-definition left PFC cathodal tDCS improved complex matchstick arithmetic task performance, which the authors attributed to the successful inhibition of previously learned rules that constrain the participants' ability to change problem representations (Luft et al., 2017).

Combining sinusoidal current stimulation with training also benefitted arithmetic performance and learning. Bilateral PFC high-frequency tRNS and rote memorization (Days 1–3) and bilateral IPS high-frequency tRNS and calculation training (Days 4–5) shortened RTs for single- and double-digit addition, subtraction, and difficult multiplication trials (with few problem repetitions) compared with easy trials (with many problem repetitions) during testing (Popescu et al., 2016). Bilateral PFC high-frequency tRNS coupled with algorithm-based calculation and memory retrieval-based drill training for 5 consecutive days also shortened RTs in both tasks and elicited sustained calculation improvement 6 months after training (Snowball et al., 2013). Left PFC theta tACS and 25 min arithmetic operation training of procedural and fact knowledge also accelerated the decrease in calculation times for fact knowledge (Mosbacher et al., 2021). On the other hand, nontraining studies indicate that bilateral PFC high-frequency tRNS is more effective during actual numerical performance testing than learning, as shown by the increased distractor distance effect in the easy and difficult trials of a double-digit addition verification task (Bieck et al., 2018). Meanwhile, in a double-digit subtraction verification task, bilateral PFC or IPS high-frequency tRNS shortened RTs during stimulation (online) and increased accuracy 7 d after stimulation (off-line; Pasqualotto, 2016).

There are also reports of null and state-dependent effects of frontal lobe stimulation on arithmetic performance. Left PFC anodal tDCS or alpha tACS did not affect learning novel arithmetic procedures and facts (Mosbacher et al., 2021), while concurrent right MFG and left IPS individually tuned HD theta tACS did not improve one-digit addition and two-digit subtraction task performance (Zhang et al., 2022). Interestingly, bilateral PFC full-spectrum tRNS failed to enhance the performance of a calculation prodigy in a complex multiplication task and reduced task accuracy of postgraduate students with highly standardized mathematics scores in simplified multiplication problems, indicating ineffective or deleterious effects for individuals with already high-performing arithmetic functioning (Krause et al., 2019). Correspondingly, anode-left and cathode-right PFC tDCS prolonged and shortened RTs for simple arithmetic decision tasks in low- and high-math anxiety individuals, respectively (A Sarkar et al., 2014).

#### Temporal cortex

The temporal cortex, including the AG, is part of the language-processing regions of the left hemisphere and serves as an additional storage of semantic knowledge (Andres et al., 2011; Siemann and Petermann, 2018). The AG is crucial for verbally mediated arithmetic fact retrieval, as demonstrated in a patient with left parietotemporal hemorrhage showing selective acalculia for addition, multiplication, and division but an intact ability to subtract (Lampl et al., 1994), and in a patient with a left superior temporal cortex lesion exhibiting a severe selective impairment in multiplication and division tasks (van Harskamp et al., 2005). In healthy participants, virtual lesions induced by 5 Hz rTMS elicited a higher error rate when applied on the left anterior STG (35%) than on the right posterior STG (8%) for simple addition tasks (Maurer et al., 2015). Meanwhile, bilateral earlobe tPCS did not affect accuracy for simple (e.g., 7 − 4) and difficult (e.g., 844 − 385) subtraction problems, ruling out the possibility that the temporal cortex performs online calculation procedures (Morales-Quezada et al., 2015).

#### Cerebellum

The cerebellum is crucial in developing early mathematical ability because of its role in sequence or pattern detection and optimization through constant error correction (Vandervert, 2017). In arithmetic performance in adults, the cerebellum is associated with various verbal working memory processes, including automated number manipulation, as reflected by the increase in BOLD signal at the cerebellar cortex and prefrontal and parietal cortices during PASAT task performance (Hayter et al., 2007). In the only NIBS study that stimulated the cerebellum, right cerebellar cathodal tDCS increased accuracy and decreased mean verbal RT and variability in the PASST task but not the PASAT task (Pope and Miall, 2012). The authors argue that these results are consistent with the notion that inhibiting the cerebellum or disinhibiting the PFC can help release cognitive resources needed for more difficult tasks (Pope et al., 2015).

### NIBS studies in patient groups

In clinical populations, the results of few arithmetic studies were somewhat inconsistent (Table 3). In a brain tumor patient, 5 Hz rTMS neuronavigation (nrTMS)-based preoperative cortical mapping of simple arithmetic processing elicited the highest error ratio for one-digit addition at the left posterosuperior frontal gyrus and left AG, partially supporting the second TCM assumption. In contrast, the highest error ratio for multiplication, subtraction, and division was elicited in frontal lobe areas, indicating reliance on working memory processes. Importantly, the resection of nrTMS arithmetic-positive cortical sites disrupted arithmetic skills as measured by the standardized number-processing and calculation task (NPCT; Ille et al., 2018). In the case of two individuals with developmental dyscalculia, left anode–right cathode bilateral PPC tDCS coupled with numerical task training (Day 1, artificial digit task with Gibson figures; Days 2–6, artificial digit task, numerical Stroop and number line task) led to numerical Stroop and number line task performance improvements during retesting (Iuculano and Cohen Kadosh, 2014). Although arithmetic proficiency was not measured directly here, this study clarifies the PPC’s and probably the IPS's function in manipulating numerical representations crucial for solving complex arithmetic problems.

Two other studies reported null results. In depression patients, although 2 week A-PASAT training (five sessions per week) with bilateral PFC tDCS (left anode–right cathode) reduced maladaptive ruminative thoughts, it failed to affect depression severity and arithmetic performance (Vanderhasselt et al., 2015). Meanwhile, in a patient with cerebral hypoxic ischemia-induced bilateral parietal lesions and Gerstmann syndrome, left PFC anodal tDCS immediately improved phonemic and semantic fluency (Sreeraj et al., 2019). Six months after hospital discharge, a 10 d consecutive left PPC anodal tDCS improved several cognitive task performances (Table 3), but not one-digit multiplication and division and all three-digit arithmetic task performances (Sreeraj et al., 2019).

## Discussion

The present review was compiled to test the TMC assumptions against the results of 37 arithmetic studies that stimulated the IPS, AG, and PSPL, as well as other cortical areas with TMS and tES. Here, we will fit the overall results with each assumption, determine whether they support them, address results inconsistencies, and give a conclusion.

### The main TCM assumptions

The reported study results corroborate the first assumption because complex arithmetic operations thought to be solved using quantity manipulations were modulated by IPS TMS (Andres et al., 2011; Salillas et al., 2012; Montefinese et al., 2017; Fresnoza et al., 2020), tDCS (Hauser et al., 2013; Kasahara et al., 2013; Klein et al., 2013a,b; Houser et al., 2014, 2015; Artemenko et al., 2015) and high-frequency tRNS stimulation (Pasqualotto, 2016; Popescu et al., 2016). TMS studies also hinted at the right IPS dominance for online calculation (Salillas et al., 2012; Montefinese et al., 2017; Fresnoza et al., 2020), which might be explained by a disruption of not only the right-lateralized ventral spatial attention system in charge of automatic orienting but also the right component of the bilateral spatial attention system in charge of intentional orienting (Corbetta and Shulman, 2002; Wassermann et al., 2008; Chica et al., 2011). A few results indicate that the IPS is involved in arithmetic fact retrieval (Andres et al., 2011; Salillas et al., 2012; Mosbacher et al., 2021). However, solving rote-learned single-digit subtraction and multiplication problems was unimpaired by left IPS 1 Hz rTMS (Fresnoza et al., 2020). Other studies showed a disruption of not only subtraction but also difficult multiplication problems (Andres et al., 2011; Salillas et al., 2012). In one case, the left IPS was not directly stimulated (Göbel et al., 2006). The null results (Hauser et al., 2016; Mosbacher et al., 2020; Zhang et al., 2022), on the other hand, support the idea that tDCS and tACS techniques have more pronounced effects only when combined with arithmetic training paradigms (Snowball et al., 2013; Pasqualotto, 2016; Popescu et al., 2016; Looi et al., 2017). There was also no evidence that full-spectrum tRNS facilitated transfer effects from an unrelated training task to arithmetic performance (Cappelletti et al., 2013, 2015).

Concerning the second TCM assumption, the reviewed studies provide mixed results. Single-digit addition and multiplication performance disruption by left AG rTMS (Maurer et al., 2015; Fresnoza et al., 2020) and tDCS (Clemens et al., 2013) provided evidence in favor of the second TCM assumption. In contrast, the disruption of complex arithmetic performance does not support the assumed role of the left AG in arithmetic fact retrieval (Göbel et al., 2006; Grabner et al., 2015; Rütsche et al., 2015; Fresnoza et al., 2020). It could be argued that complex arithmetic problems are solvable by retrieval, namely, by breaking them down into simpler, retrievable problems (Dehaene and Cohen, 1995; Fayol and Thevenot, 2012; Siemann and Petermann, 2018). However, some new theories offer further explanations. The symbol-to-referent mapping hypothesis suggests that the left AG can match quantities to Arabic digits (Price and Ansari, 2011; Bloechle et al., 2016), while the attention to memory model proposes that the left AG is a flexible interface that adjusts and adapts attentional demands: top-down for magnitude manipulations and bottom-up for fact retrieval (Cabeza et al., 2008, 2012; Klein et al., 2013a,b; Bloechle et al., 2016). In other words, the AG could subserve fact retrieval for rote-learned arithmetic problems and online calculation for complex problems. The only drawback is that participant's trial-by-trial strategies are typically not assessed to confirm such an assumption.

For the third assumption, namely, that the PSPL, bilaterally, plays a role in arithmetic, two studies showed the absence of bilateral PSPL rTMS effect on single-digit addition, subtraction, multiplication, and division task performance (Andres et al., 2011; Maurer et al., 2015). This indicated that the PSPLs have no obligatory role in arithmetic processing and may only aid numeric computations by serving as a visuospatial medium for estimations (Chochon et al., 1999; Nieder and Dehaene, 2009) or by orienting and shifting attention toward the left or right side of a mental numerical continuum, helpful for the intuition of removing or adding quantities (Dehaene et al., 2003; Knops et al., 2009; Andres et al., 2011). So far, there is insufficient evidence for a definitive answer and that the third assumption requires further investigation.

### Other cortical targets

The close examination of studies involving brain areas excluded from the main TCM assumptions showed that they also contribute to arithmetic processing. Results suggest the involvement of the right SMG for problems requiring online calculation (Maurer et al., 2015; Montefinese et al., 2017) and the left SMG for problems that require retrieval of answers from long-term memory (Göbel et al., 2006; Klichowski and Kroliczak, 2020). These result patterns are consistent with the meta-analysis of neuroimaging studies showing a clear link between the left SMG and arithmetic facts, as well as an association between the right SMG and approximate nonsymbolic magnitude processing (Faye et al., 2019). Interestingly, the interhemispheric differences in the SMG function were similar to those of the AG (Montefinese et al., 2017). It could be that the anatomical proximity of the SMG and AG is responsible for these results.

The evidence from frontal lobe stimulation points to three key findings. First, PFC stimulation reduces the impact of negative emotions, anxiety, distractors, and previously learned rules on arithmetic performance (Sarkar et al., 2014; Plewnia et al., 2015; Luft et al., 2017; Bieck et al., 2018). The consensus is that PFC stimulation reduces interference from these impediments, preserves working memory resources, and reduces attentional biases. Second, the stimulation has different effects on arithmetic performance for different groups of individuals. Bilateral PFC full-spectrum tRNS impairs individuals with high-performing arithmetic skills (Krause et al., 2019), while bilateral PFC tDCS improves and impairs the performance of individuals with high or low mathematics anxiety, respectively (Sarkar et al., 2014).

Third, the PFC, mainly the dorsolateral region, supports complex arithmetic task performance. It has been shown that left PFC tDCS (Gill et al., 2015; Plewnia et al., 2015; Pope et al., 2015; Luft et al., 2017; Mosbacher et al., 2020), as well as bilateral PFC high-frequency tRNS with training (Snowball et al., 2013; Popescu et al., 2016) or without training (Pasqualotto, 2016; Bieck et al., 2018), had beneficial effects on complex arithmetic task performance. These results are consistent with the idea that complex calculations have a higher demand for domain-general processes, including working memory (e.g., to maintain intermediate results) and attention, as they involve numerical decision-making and require many steps to generate a correct answer (Arsalidou and Taylor, 2011; Siemann and Petermann, 2018; Klichowski and Kroliczak, 2020). A hierarchical organization of the frontal gyri in arithmetic was also proposed. The inferior frontal gyrus is assumed to be involved in processing simple numerical tasks with few storage or procedural requirements, the middle frontal gyri in managing several cognitive procedural steps (e.g., carrying a number in two-digit addition), and the medial and superior frontal gyri in generating strategies to solve complex multistep problems (Arsalidou and Taylor, 2011).

The role of the temporal cortex and cerebellum in arithmetic warranted further explorations. The evidence from a few studies reported here suggests a role for the left temporal cortex in arithmetic fact retrieval since 5 Hz rTMS of the left anterior STG induced a high error rate for a simple addition (Maurer et al., 2015) and that tPCS did not affect simple and difficult subtraction problems that require online calculation procedures (Morales-Quezada et al., 2015). Meanwhile, the only study that stimulated the right cerebellum with cathodal tDCS revealed its supportive role in domain-general processes such as working memory, particularly for cognitively demanding arithmetic tasks such as PASST (Pope and Miall, 2012).

### Limitations of the literature

The reviewed articles have potential limitations that must be mentioned. First, suitable control conditions are missing in some studies. This means that the domain specificity of the results for arithmetic is unclear. Second, divergent results can be linked to task heterogeneity. Subtraction and multiplication are commonly used to tap into the brain networks subserving the mental manipulation of numerical quantities and arithmetic fact retrieval, respectively. This is problematic since arithmetic problems can be solved using both strategies. However, except for Fresnoza et al. (2020) and Mosbacher et al. (2020), no other study considered the impact of trial-by-trial solution strategies on arithmetic performance. Third, the modulatory effects of NIBS on cognitive function are sensitive to a range of methodological- and subject-specific factors that are rarely considered in many studies [for an extensive review, see Ridding and Ziemann (2010), Paulus et al. (2016), Sarkar and Cohen Kadosh (2016), Silvanto and Cattaneo (2017), and Hartwigsen and Silvanto (2023)]. For instance, stimulation intensities are typically set to values known to inhibit and facilitate corticospinal excitability. However, studies have shown that distinct TMS intensity ranges can induce distinct effects: low intensities facilitate neural activity and behavior, whereas high intensities induce suppression (Silvanto and Cattaneo, 2017). Furthermore, these ranges are shifted by changes in neural excitability. For instance, a TMS intensity, which normally induces suppression, can have a facilitatory effect if the stimulated neurons are being inhibited by ongoing task-related processes or preconditioning (Silvanto and Cattaneo, 2017). Therefore, stimulation intensity and brain-state interactions could explain why NIBS methods' distinction as either inhibitory or facilitatory does not always map exactly onto the respective behavioral outcomes (Hartwigsen and Silvanto, 2023).

The notion that NIBS does not work for everyone can also be accounted for by interindividual variability in the response to stimulation protocols. For instance, anodal tDCS only increased corticospinal excitability in individuals who are sensitive to single-pulse TMS (Labruna et al., 2016). Similarly, anodal tDCS improved response inhibition (measured by a stop signal task) in individuals with high brain connectivity but not in those with low connectivity (Li et al., 2019). On the other hand, the impact of left PFC cathodal tDCS was predicted by the baseline GABA–glutamate ratio. Individuals with higher level of inhibition (more GABA relative to glutamate) experienced more disruption in response selection paradigms (Filmer et al., 2019). It is interesting to note that the effect of anodal tDCS is also influenced by attention allocation. Participants who were engaged in a cognitive task during motor cortex stimulation did not experience an increase in corticospinal excitability (Antal et al., 2007). This highlights the need to identify and control factors that may affect the response to NIBS. While controlling the neuronal state is difficult, priming measures and ensuring participants are attentive yet relaxed can help reduce outcome variations. However, factors such as age, gender, and genetic polymorphisms cannot be changed, so controlling them through careful experimental design is crucial (Huang et al., 2017).

Lastly, the lack of tES focality is another limitation in terms of site-specific targeting. TES effects are limited not only to cortical areas beneath the electrodes but also to areas affected by the electric field generated between them, even reaching deep regions (Bikson et al., 2010; Garcia-Sanz et al., 2022). Out of all the analyzed tES studies, only seven conducted electric field distribution simulations (Klein et al., 2013a,b; Artemenko et al., 2015; Grabner et al., 2015; Hauser et al., 2016; Luft et al., 2017; Bieck et al., 2018; Zhang et al., 2022). This means that in the remaining studies, the target areas may not be adequately reached and optimally stimulated, specifically during IPS stimulation, where the depth of current penetration is critical. Nevertheless, the studies that did use finite-element method-based electric field calculations showed activation peaks in the IPS higher than what is recommended (0.017 mA/cm^2^) to modify cortical excitability by tDCS in humans (Klein et al., 2013a,b; Artemenko et al., 2015; Hauser et al., 2016; Bieck et al., 2018; Zhang et al., 2022). It is essential for future studies to perform subject-specific electric field simulation to select the optimal current dose and pinpoint cortical areas that are likely to be activated by tES (Kashyap et al., 2021; Van Hoornweder et al., 2023). Techniques such as transcranial-focused ultrasound, transcranial temporal interference stimulation, and transcutaneous vagus nerve stimulation that offer superior spatial resolution and penetration depth compared with conventional stimulation paradigms could also potentially resolve the focality issue in future studies. Machine learning methods (e.g., personalized Bayesian optimization) can also be used to search, learn, and recommend neurostimulation parameters based on a participant's baseline arithmetic abilities (van Bueren, 2021b). Most importantly, further studies are warranted to better understand putative mechanisms underlying the effect of NIBS methods. This will help validate inferences on the causal role of a cortical area for arithmetic with absolute certainty.

## Conclusions

This review provides a new perspective on TCM based on the results of arithmetic processing modulated by NIBS methods. Taken together, the results yielded three main conclusions. First, overall findings corroborated the first assumption of the TCM that the bilateral IPS is involved in numerical quantity manipulation. Second, the second assumption of the TCM that the left AG has an exclusive role in arithmetic fact retrieval cannot be confirmed because of mixed results. The third assumption about the role of the bilateral PSPL in the number domain could also not be supported due to the limited number of studies. Finally, collective results from studies that stimulated brain areas outside those postulated by the TCM showed that the bilateral SMG and bilateral DLPFC are also crucial for arithmetic performance. In contrast, although promising, the evidence from studies involving the temporal cortex and cerebellum is still insufficient. In summary, the available evidence from NIBS studies favors the existence of a wider cortical network rather than a one-to-one correspondence between numerical abilities and the specific parietal brain areas postulated by the TCM (Nieder, 2016).

## Synthesis

Reviewing Editor: Alexander Soutschek, Ludwig-Maximilians-Universitat Munchen Decisions are customarily a result of the Reviewing Editor and the peer reviewers coming together and discussing their recommendations until a consensus is reached. When revisions are invited, a fact-based synthesis statement explaining their decision and outlining what is needed to prepare a revision will be listed below. The following reviewer(s) agreed to reveal their identity: Philipp Schroeder.

Reviewer #1

This is a very interesting review. However, the authors should have taken into account many more issues that affect brain stimulation results, such as intensity, task difficulty, brain state (see e.g. Cattaneo & Silvanto 2017). The issue here is that if the mechanisms of brain stimulation are not considered, then various subtleties are being left out. For example, the behahvioral tasks used vary greatly; from adaption (e.g. Cohen Kadosh et al, 2010) to typical virtual lesion studies. These differenes may very well affect the conclusions drawn from individual studies (e.g. TMS at low intensities enhances but at high intensities disrupts...)

Reviewer #2

The submission „Probing our built-in calculator: a systematic narrative review of non-invasive brain stimulation studies on arithmetic operation-related brain areas" reports a systematic narrative review on neurostimulation and neuromodulation studies (using transcranial magnetic stimulation and transcranial electric stimulation) on arithmetic performance. The review is organized along the neuropsychological assumptions underlying the influential triple-code model of numerical cognition (TCM).

This is a refreshing, well structured, and informative review. Based on existing evidence, the authors conclude that the first assumption of the TCM is bolstered, whereas the evidence for assumption 2 and 3 is incomplete or mixed. The review is clearly motivated by the TCM, some of the limitations of non-invasive brain stimulation (particularly the interpretation of their effects and open fundamental questions) should be introduced in more depth. I have only small comments.

Intro:

- The TCM is nicely introduced, I find the section on non-invasive brain stimulation too shallow. In particular, the authors should introduce a) how the (neuro-)physiological consequences of NIBS would affect (numerical) cognition and b) the fact that individual variability in terms of both physiological but also psychological variables can moderate NIBS outcomes

- The IPS as a core structure in numerical cognition is a relatively small region, relatively deeply situated in the cortex. Neuromodulation with TMS and tES, in contrast, is able to modulate cortical areas. How can the authors be sure that stimulation in the reviewed studies actually reaches IPS?

Methods:

- Please share the PRISMA checklist as supplementary material with the submission

- Was the review, its search strategy and extraction procedure preregistered?

Results:

- Regarding tRNS, please indicate whether high-frequency or low-frequency tRNS was applied.

- Regarding prefrontal stimulation, the authors are inconsistent in their terminology across sections. Studies are referred to "prefrontal cortex" in their results section, whereas they more specifically claim that "dorsolateral prefrontal cortex" was stimulated in their discussion section. Given that the spatial precision in most of the studies was low, especially using tDCS, I would suggest to rather refer in the text to "prefrontal cortex", which might also include other parts than DLPFC.

Discussion:

- Could emerging non-invasive brain stimulation methods such as tFUS, tTIS, or even tVNS contribute to the open questions in the field of numerical cognition?

- In their limitations sections, the authors correctly criticize the lack of focality of tES. However, using oppositional placements or high-definition electrode montages does not per se solve this issue. Electric field modeling and personalized, individualized stimulation could help, but on top of that, more fundamental research into the mechanisms underlying NIBS (variability) is still required.

## Author Response

Reviewer #1 This is a very interesting review. However, the authors should have taken into account many more issues that affect brain stimulation results, such as intensity, task difficulty, brain state (see e.g. Cattaneo & Silvanto 2017). The issue here is that if the mechanisms of brain stimulation are not considered, then various subtleties are being left out. For example, the behavioral tasks used vary greatly; from adaption (e.g. Cohen Kadosh et al, 2010) to typical virtual lesion studies. These differences may very well affect the conclusions drawn from individual studies (e.g. TMS at low intensities enhances but at high intensities disrupts...).

Response: We appreciate the valuable feedback provided by the reviewer regarding the various factors that can influence the impact of non-invasive brain stimulation (NIBS) techniques on cognitive functions. We also acknowledge that our previous explanation of the NIBS mechanisms was overly simplistic. Therefore, in this revised version of the manuscript, we have included a more detailed discussion on the online and offline effects of TMS and tES (highlighted in blue), as well as their potential interactions with task-related neural processes. This discussion can be found in the Introduction section, specifically on pages 5-10.

We have also made significant improvements to the limitations section (Pages 26-28) of our review paper. The relevant text is highlighted in blue, and now includes a brief yet comprehensive discussion of the factors that may limit the interpretation of the overall results. These factors include stimulation intensities, interindividual variability, and the focality of tES methods. For a more detailed discussion of other factors that may affect brain stimulation effects, we recommend that readers refer to the review papers (below) which were cited on page 26.

Ridding, M.C. & Ziemann, U. (2010) Determinants of the induction of cortical plasticity by non-invasive brain stimulation in healthy subjects. J Physiol, 588, 2291-2304.

Sarkar, A. & Cohen Kadosh, R. (2016) Transcranial electrical stimulation and numerical cognition. Canadian Journal of Experimental Psychology/Revue canadienne de psychologie expérimentale, 70, 41-58.

Hartwigsen, G. & Silvanto, J. (2023) Noninvasive Brain Stimulation: Multiple Effects on Cognition. The Neuroscientist, 29, 639-653.

Silvanto, J. & Cattaneo, Z. (2017) Common framework for "virtual lesion" and state-dependent TMS: The facilitatory/suppressive range model of online TMS effects on behavior. Brain Cogn, 119, 32-38.

Paulus, W., Nitsche, M.A., & Antal, A. (2016) Application of Transcranial Electric Stimulation (tDCS, tACS, tRNS). Eur Psychol, 21, 4-14.

Reviewer #2 The submission „Probing our built-in calculator: a systematic narrative review of non-invasive brain stimulation studies on arithmetic operation-related brain areas" reports a systematic narrative review on neurostimulation and neuromodulation studies (using transcranial magnetic stimulation and transcranial electric stimulation) on arithmetic performance. The review is organized along the neuropsychological assumptions underlying the influential triple-code model of numerical cognition (TCM).

This is a refreshing, well structured, and informative review. Based on existing evidence, the authors conclude that the first assumption of the TCM is bolstered, whereas the evidence for assumption 2 and 3 is incomplete or mixed. The review is clearly motivated by the TCM, some of the limitations of non-invasive brain stimulation (particularly the interpretation of their effects and open fundamental questions) should be introduced in more depth. I have only small comments.

1. Introduction: The TCM is nicely introduced, I find the section on non-invasive brain stimulation too shallow. In particular, the authors should introduce a) how the (neuro-) physiological consequences of NIBS would affect (numerical) cognition and b) the fact that individual variability in terms of both physiological but also psychological variables can moderate NIBS outcomes Response: We would like to express our gratitude to the reviewer for providing us with these valuable points. We apologize for the overly simplistic discussion of non-invasive brain stimulation (NIBS) methods in the introduction section. In order to address this issue, we have made significant changes to the "Non-invasive brain stimulation (NIBS)" section from Pages 5-6. We have included the proposed online and offline mechanism of action of TMS and tES methods and provided a concise discussion on how these mechanisms would affect numerical cognition.

We have decided to address the reviewer's second point by incorporating it into the improved "Limitations of the Literature" section on pages 26-28. We have added interindividual variability in response to stimulation protocols as a limitation and have mentioned studies, such as Li et al. (2019) on functional connectivity and Filmer et al. (2019) on the baseline concentration of GABA and glutamate as examples of physiological variables, and Antal et al. (2007) on attentional state, as example of psychological variables that can impact the response to NIBS.

Li, L.M., Violante, I.R., Leech, R., Hampshire, A., Opitz, A., McArthur, D., Carmichael, D.W., & Sharp, D.J. (2019) Cognitive enhancement with Salience Network electrical stimulation is influenced by network structural connectivity. Neuroimage, 185, 425-433 Filmer, H.L., Ehrhardt, S.E., Bollmann, S., Mattingley, J.B., & Dux, P.E. (2019) Accounting for individual differences in the response to tDCS with baseline levels of neurochemical excitability. Cortex, 115, 324-334.

Antal, A., Terney, D., Poreisz, C., & Paulus, W. (2007) Towards unravelling task-related modulations of neuroplastic changes induced in the human motor cortex. European Journal of Neuroscience, 26, 2687-2691.

2. The IPS as a core structure in numerical cognition is a relatively small region, relatively deeply situated in the cortex. Neuromodulation with TMS and tES, in contrast, is able to modulate cortical areas. How can the authors be sure that stimulation in the reviewed studies actually reaches IPS? Response: We fully agree with the reviewer's point regarding IPS stimulation, in particular the depth of current penetration. Although both TMS and tES suffer from depth-focality tradeoffs, this issue is less critical for the reviewed TMS studies because the figure-of-eight coils' electric field penetration is 0.9-3.4 cm (Deng et al., 2013). Therefore, given the average scalp-cortical distance of 1.5 cm, TMS stimulation of the IPS is feasible. In addition, the majority of reviewed TMS studies used fMRI data-based neuronavigation which ensures focal stimulation.

Concerning tES, current flow, depth of penetration, and peak density in the cortex are difficult to know without a simulation study prior to the experiment. This issue was added in the limitation section because out of all the analyzed tES studies, only five conducted electric field distribution simulations for the IPS (Klein et al., 2013; Artemenko et al., 2015; Hauser et al., 2016; Bieck et al., 2018; Zhang et al., 2022), one for left AG (Grabner et al., 2015) and one for the DLPFC (Luft et al., 2017). Nevertheless, studies involving the IPS through current density simulation, showed that tES can induce activation peaks in the IPS higher than what is recommended (0.017 mA/cm²) to modify cortical excitability by tDCS in humans. tDCS stimulation (1 mA) induced a field intensity of 0.23 V/m in Klein et al. (2013), 0.019 mA/cm2 in Artemenko et al. (2015) in the bilateral intraparietal cortices, while in Hauser et al. (2016) tDCS current density was 0.02 mA/cm2 in the left parietal cortex. Therefore, we can argue that the current from tES stimulation can reach the IPS. We also added the current simulation information on the tES table (Table 2).

Deng, Z.-D., Lisanby, S.H., & Peterchev, A. V. (2013) Electric field depth-focality tradeoff in transcranial magnetic stimulation: Simulation comparison of 50 coil designs. Brain Stimul, 6, 1-13.

3. Methods: Please share the PRISMA checklist as supplementary material with the submission - Was the review, its search strategy and extraction procedure preregistered? Response: Thank you for your suggestion. However, eNeuro does not accept supplemental material. Therefore, we will keep the PRISMA checklist as an included figure in the manuscript. Our review was not preregistered.

4. Results: Regarding tRNS, please indicate whether high-frequency or low-frequency tRNS was applied.

Response: We apologize for the missing information. The manuscript has been updated to categorize tRNS protocols into low-frequency, high-frequency, and full-spectrum tRNS.

5. Regarding prefrontal stimulation, the authors are inconsistent in their terminology across sections. Studies are referred to "prefrontal cortex" in their results section, whereas they more specifically claim that "dorsolateral prefrontal cortex" was stimulated in their discussion section. Given that the spatial precision in most of the studies was low, especially using tDCS, I would suggest to rather refer in the text to "prefrontal cortex", which might also include other parts than DLPFC.

Response: We apologize for the confusing terminology used in our previous text. Following the reviewers' advice, we used "prefrontal cortex or PFC" specifically for tES studies.

6. Discussion: Could emerging non-invasive brain stimulation methods such as tFUS, tTIS, or even tVNS contribute to the open questions in the field of numerical cognition? Response: The potential of transcranial-focused ultrasound (tFUS), transcranial temporal interference stimulation (tTIS), and transcutaneous vagus nerve stimulation (tVNS) as non-invasive research and therapeutic tools are just emerging. To our knowledge, none of these novel methods has been used to explore numerical cognition so far. However, because they have some properties that are far superior to conventional TMS and tES methods, their use in elucidating causal brain function related to number processing is promising. For instance, tFUS shows advantages in spatial resolution and penetration depth when compared with electrical or magnetic stimulation (Legon et al., 2018), making it an ideal method to stimulate cortical areas such as the IPS. tTIS also has far superior focality than tACS and can stimulate deeper brain regions without stimulating the overlaying cortex (Rampersad et al., 2019). This will be useful when targeting subcortical processing pathways for easier (verbally mediated) and more difficult (magnitude-related) mental arithmetic within the proposed fronto-parietal network for numerical cognition (Klein et al., 2013). On the other hand, the application of auricular tVNS in cognitive neuroscience is rather limited because it is applied only to the left ear. Nonetheless, there is anatomical evidence of several brainstem nuclei in the medulla oblongata, where the vagus nerve arises (locus coeruleus and spinal nucleus of the trigeminal nerve), sending projections to structures that are believed to be engaged in memory processes. These include the caudate nucleus for procedural memory that may function in performing steps to reduce a fraction or perform long division, the amygdala for emotional memory, which could be relevant in mathematical anxiety, and hippocampal formation for declarative and spatial memory that plays an important role in the retrieval of arithmetic facts or memory-based problem-solving strategies. We added a recommendation for the use of these alternative techniques in the "Limitations of the Literature" section of the manuscript on page 28.

Legon, W., Ai, L., Bansal, P., and Mueller, J. K. (2018). Neuromodulation with single-element transcranial focused ultrasound in human thalamus. Hum. Brain Mapp 39, 1995-2006. doi: 10.1002/hbm.23981 Rampersad, S., Roig-Solvas, B., Yarossi, M., Kulkarni, P.P., Santarnecchi, E., Dorval, A.D., & Brooks, D.H. (2019) Prospects for transcranial temporal interference stimulation in humans: A computational study. Neuroimage, 202, 116124.

Klein, E., Moeller, K., Glauche, V., Weiller, C., & Willmes, K. (2013) Processing Pathways in Mental Arithmetic-Evidence from Probabilistic Fiber Tracking. PLoS One, 8, e55455.

7. In their limitations sections, the authors correctly criticize the lack of focality of tES. However, using oppositional placements or high-definition electrode montages does not per se solve this issue. Electric field modeling and personalized, individualized stimulation could help, but on top of that, more fundamental research into the mechanisms underlying NIBS (variability) is still required.

Response: We appreciate the reviewer's suggestion and have made the necessary changes to the limitation section on page 28. It now states that future studies should perform subject-specific electric field simulation to select the ideal current dose and identify cortical areas that are likely to be activated by tES. Additionally, we emphasize the need for further research to gain a better understanding of the potential mechanisms underlying the effect of NIBS methods.
